# Neuroimmunoendocrine Link Between Chronic Kidney Disease and Olfactory Deficits

**DOI:** 10.3389/fnint.2022.763986

**Published:** 2022-01-31

**Authors:** Rebeca Corona, Benito Ordaz, Ludivina Robles-Osorio, Ernesto Sabath, Teresa Morales

**Affiliations:** ^1^Instituto de Neurobiología, Universidad Nacional Autónoma de México, Querétaro, Mexico; ^2^Facultad de Nutrición, Universidad Autónoma de Querétaro, Querétaro, Mexico

**Keywords:** chronic kidney disease, olfactory deficit, neuroinflammation, prolactin, olfactory epithelium, olfactory bulb

## Abstract

Chronic kidney disease (CKD) is a multifactorial pathology that progressively leads to the deterioration of metabolic functions and results from deficient glomerular filtration and electrolyte imbalance. Its economic impact on public health is challenging. Mexico has a high prevalence of CKD that is strongly associated with some of the most common metabolic disorders like diabetes and hypertension. The gradual loss of kidney functions provokes an inflammatory state and endocrine alterations affecting several systems. High serum levels of prolactin have been associated with CKD progression, inflammation, and olfactory function. Also, the nutritional status is altered due to impaired renal function. The decrease in calorie and protein intake is often accompanied by malnutrition, which can be severe at advanced stages of the disease. Nutrition and olfactory functioning are closely interconnected, and CKD patients often complain of olfactory deficits, which ultimately can lead to deficient food intake. CKD patients present a wide range of deficits in olfaction like odor discrimination, identification, and detection threshold. The chronic inflammatory status in CKD damages the olfactory epithelium leading to deficiencies in the chemical detection of odor molecules. Additionally, the decline in cognitive functioning impairs the capacity of odor differentiation. It is not clear whether peritoneal dialysis and hemodialysis improve the olfactory deficits, but renal transplants have a strong positive effect. In the present review, we discuss whether the olfactory deficiencies caused by CKD are the result of the induced inflammatory state, the hyperprolactinemia, or a combination of both.

## Introduction

The kidneys are in charge of filtering and eliminating the metabolic products and toxins from the blood, maintaining the control of the extracellular fluid, and the electrolyte and acid-base balance (Dhondup and Qian, 2017). Chronic kidney disease (CKD) is a major public health problem affecting approximately 9–12% of the population worldwide (Ji-Cheng and Lu-Xia, 2019; Cockwell and Fisher, 2020). In Mexico, diabetes and hypertension, two chronic metabolic diseases, are associated with CKD; studies on the latter’s prevalence and incidence are scarce (Gutierrez-Padilla et al., 2010; Aguilar and Madero, 2019).

## Chronic Kidney Disease

**FIGURE 1:**
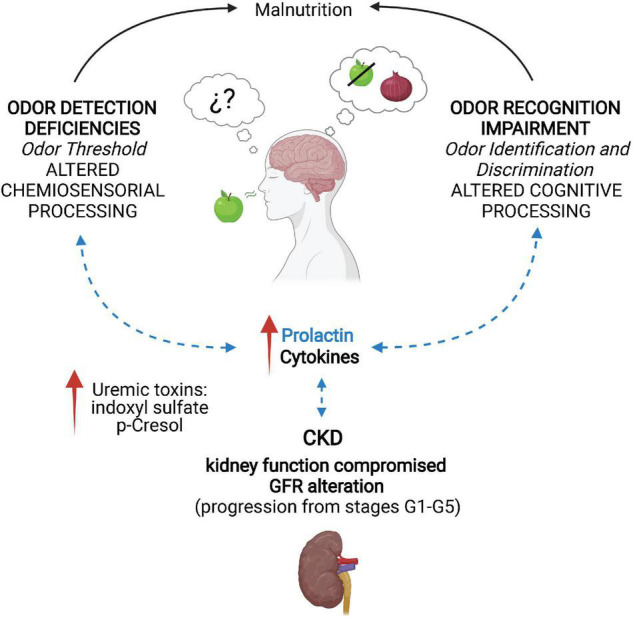
Neuroimmune alterations and olfactory deficiencies promoted by chronic kidney disease. In chronic kidney disease (CKD), kidney function is compromised due to alterations of the glomerular filtration rate (GFR), as the disease progresses from stage G1 (≥90 ml/min/1.73 m^2^) to G5 (<15 ml/min/1.73 m^2^). This leads to increased levels of the uremic toxins indoxyl sulfate and p-Cresol. The toxin accumulation activates the immune system by releasing cytokines and increasing prolactin (PRL) levels due to inadequate GFR. CKD patients develop deficiencies in odor detection, identification, and discrimination that are reversible after renal transplantation. Additionally, olfactory alterations can cause malnutrition, worsening the patients’ condition. Evidence on the regulatory role of PRL in olfactory processing suggests an interaction between CKD-induced high PRL levels and the olfactory deficiencies (dotted blue arrows). Created with BioRender.com.

CKD is defined as structural and functional abnormalities in the kidney for more than 3 months, mainly caused by deficient glomerular function (Figure 1; No Author, 2013; Levey et al., 2020). The glomerular filtration rate (GFR) is used as an overall index of kidney function. CKD is classified to facilitate the diagnosis and treatment of patients with kidney damage and is based on an estimated GFR for three consecutive months. The classification is divided into five stages (G1–G5): normal to mildly decreased GFR (G1–G2; ≥ 90 and 60–89 ml/min/1.73 m^2^, respectively), moderately to severely decreased GFR (G3–G4; 30–59 and 15–29 ml/min/1.73 m^2^, respectively) and kidney failure (G5; < 15 ml/min/1.73 m^2^) (Levey et al., 2020). When GFR is < 15 ml/min/1.73 m^2^ (G5), patients are diagnosed with end-stage kidney disease (ESKD) and usually require a renal replacement therapy that could be dialysis or kidney transplantation (Krajewska et al., 2020).

CKD leads to uremia, a condition induced by the accumulation of uremic toxins in the body (Cigarran Guldris et al., 2017). Classically, the uremic toxins are classified based on their physicochemical characteristics into small water-soluble compounds, protein-bound, and middle molecules (Cobo et al., 2018; Vanholder et al., 2018). Uremic toxin retention negatively interacts with a variety of biological systems, and the progression of CKD to ESKD is strongly associated with the accumulation of uremic metabolites in the blood, with concentrations that reach 10–100-fold in CKD patients compared to the healthy population (Bobot et al., 2020).

The origin of uremic toxins in CKD is manifold. In CKD, dysbiosis in the intestinal microflora with increased pathogenic flora occurs (Cigarran Guldris et al., 2017). Uremic toxins are generated through protein fermentation by colonic microbiota (Rysz et al., 2021). Proteins degraded in the colon are broken down by intestinal bacteria to metabolites, such as phenols and indoles (Cigarran Guldris et al., 2017), and subsequently eliminated in the feces. However, some of them are absorbed and eliminated by the kidney (Cigarran Guldris et al., 2017).

In CKD, the uremic toxins generated by the intestinal microflora are protein-bound indoles and phenols (Rysz et al., 2021). Indole is a metabolite of the dietary amino acid L-tryptophan after fermentation by intestinal bacteria, which is rapidly absorbed by intestinal epithelial cells and subsequently sulfated to indoxyl sulfate (IS) in the liver (Cigarran Guldris et al., 2017). IS directly induces apoptotic and necrotic cell death of tubular cells, increases oxidative stress and the production of transforming growth factor β1 (TGF-β1), decreases the intracellular glutathione, and promotes renal fibrosis with the consequent decline in the kidney function (Figure 1; Cheng et al., 2020). The most studied phenolic uremic toxin is p-Cresol, which is generated by the intestinal microbial breakdown of tyrosine/phenylalanine and causes endothelial dysfunction and vascular calcification among other effects (Opdebeeck et al., 2019). In proximal tubular cells, it increases NADPH activity, mRNA levels of inflammatory cytokines, and the secretion of TGF-β1 (Watanabe et al., 2013). P-Cresol is associated with renal fibrosis and progression of CKD (Figure 1; Watanabe et al., 2013; Opdebeeck et al., 2019).

Oxidative stress and chronic inflammation have a central role in the pathophysiological process of uremia, causing secondary complications in various systems, among them the central nervous system (CNS) (Watanabe et al., 2014; Tsuruya and Yoshida, 2018). More specifically, patients commonly present neurological disorders such as restless leg syndrome (Safarpour et al., 2020), dementia, stroke (Bugnicourt et al., 2013), and uremic encephalopathy (Olano et al., 2021). In animal models, chronic exposure to IS promotes its accumulation in the brain stem, reducing local monoamine levels and leading to reduced locomotor and exploratory activity (Karbowska et al., 2020). IS promotes the expression of oxidative stress markers and inflammation in glial cells compromising their functioning and leading to neurodegeneration (Adesso et al., 2017).

CKD is characterized by a low-grade systemic inflammatory status that plays a key role in the progression of the disease and the increased morbidity and mortality (Mihai et al., 2018), which are also affected by malnutrition and chronic inflammation (Tinti et al., 2021). As illustrated in Figure 1, CKD is associated with the dysregulation of synthesis, release, and degradation of soluble molecules of the immune system, the disruption of cytokines and inflammatory mediators, and decreased adrenal clearance accounting for high levels of circulating cytokines (Rossaint et al., 2016). Inflammatory processes are highly influenced by sex hormones, with males being more susceptible to exacerbation of CKD (Kang et al., 2014; Franco-Acevedo et al., 2021).

### Endocrine System and Chronic Kidney Disease

Sex and gender disparities exists in CKD: the worldwide data analysis of the 2016 Global Burden of Disease, Injuries, and Risk Factors Study revealed that the CKD prevalence is higher in women than in men (Bikbov et al., 2018). However, the CKD progression and risk of death is lower in women, while men tend to escalate more rapidly to ESKD (Brar and Markell, 2019). In this vein, as pointed out in a recent review by Machluf et al. (2020), sex differences and their relationship to CKD incidence are complex, and various factors, such as age, ethnicity, lifestyle habits, and access to health care, should be considered (Mills et al., 2015; Bikbov et al., 2018; Carrero et al., 2018; Machluf et al., 2020).

The deficient and progressive GFR leads to renal damage and CKD with some sex differences explaining part of the sex disparities (Brar and Markell, 2019). For instance, men have larger and heavier kidneys compared to women (Miletić et al., 1998; Neugarten et al., 2002; Sabolic et al., 2007; Franco-Acevedo et al., 2021; Lima-Posada and Bobadilla, 2021). In rodents, males develop glomerular hypertrophy, tubular fibrosis, proteinuria, and enhanced oxidative stress after kidney ischemia-reperfusion injury, while females do not present CKD-related deterioration, suggesting that testosterone and estrogen may contribute to the susceptibility and progression of the disease (Lima-Posada et al., 2017). Furthermore, gonadectomized males presented reduced inflammation with decreased expression of pro-inflammatory cytokines, and testosterone replacement exacerbated the inflammatory response. However, in females, ovariectomy aggravated the inflammation but treatment with estrogen did not completely revert the effect (Kang et al., 2014).

It is not surprising that sex hormones influence kidney function since androgen, estrogen, and progesterone receptors exist in this organ (Davidoff et al., 1980; Takeda et al., 1990; Bhat et al., 1993; Han et al., 2001; Sabolic et al., 2007). In humans, sex hormones are also produced within the kidney, raising the possibility of autocrine and paracrine regulation of renal functions (Sharpe, 1998; Quinkler et al., 2003). Locally produced sexual hormones activate their receptors even in the absence of circulating hormones (Martucci and Fishmann, 1993; Dalla Valle et al., 2004). Furthermore, several studies on CDK show its association with elevated concentrations of prolactin (PRL) (Figure 1; Franco-Acevedo et al., 2021).

#### Prolactin and Chronic Kidney Disease

PRL is a peptide hormone synthesized in the anterior pituitary gland and released into the systemic circulation to carry out more than 300 biological actions: it is associated with the reproductive system, including the classical lactogenic action in the mammary gland, osmoregulation, immune response, brain function, and behavior (Ben-Jonathan et al., 1996). The PRL receptor is a member of the class I cytokine-hematopoietin receptor superfamily, widely expressed in different systems (Ma et al., 2005). Within the CNS, PRL actions include the control of its secretion (Freeman et al., 2000), neurogenesis in the olfactory bulb (Shingo et al., 2003), and neuroprotection (Morales, 2011), among others. The secretion of PRL from the pituitary is regulated by the inhibitory action of dopamine, released from the tuberoinfundibular (TIDA) neurons of the arcuate nucleus (Freeman et al., 2000). Dopamine tonically inhibits the secretion of PRL (Demaria et al., 2000) through the short feedback loop between lactotrophs and TIDA neurons, which is largely responsible for PRL homeostasis (Grattan et al., 2001).

The PRL regulatory loop might be disrupted in CKD since elevated concentrations of PRL or hyperprolactinemia are present in ∼30% of the patients in the early stages and 60–80% in the advanced stages of CKD (Lo et al., 2017; Dourado et al., 2020). To date, there is no evidence on whether hyperprolactinemia is caused by deficient metabolic depuration or the central dysregulation of PRL secretion (Carrero et al., 2012; Gungor et al., 2013), but deleterious effects of hyperprolactinemia are known to include endocrine, metabolic, and immune effects (Borba et al., 2018).

As for the systemic accumulation of PRL and its inadequate clearance related to deficient renal filtration, there is some evidence to consider. This hormone participates in osmoregulation, principally in salt and water metabolism (Loretz and Bern, 1982). Expression of the PRL gene and the binding of the hormone have been identified in the Bowman’s capsule and proximal tubule cells of the kidney (Sakai et al., 1999). A large percentage of PRL in the renal artery is metabolized and eliminated by the kidneys (Emmanouel et al., 1981). PRL reduces sodium, potassium, and water excretion (Stier et al., 1984), while unilateral nephrectomy and water deprivation significantly increase serum PRL levels (Horrobin et al., 1971; Loretz and Bern, 1982; Franco-Acevedo et al., 2021).

The PRL receptor is also expressed in immune system cells, including lymphocytes, macrophages, and thymic epithelial cells (Bouchard et al., 1999), where the ligand acts as an immunomodulatory cytokine (Leite De Moraes et al., 1995). Hyperprolactinemia is associated with autoimmune diseases such as lupus and multiple sclerosis (Borba et al., 2018) and possibly contributes to the inflammatory mechanisms associated with CKD (Tinti et al., 2021).

A recent study addressing the consequences of elevated PRL in CKD proposed that the diagnosis of hyperprolactinemia in this population is difficult and dialysis therapies, such as conventional hemodialysis and peritoneal dialysis, do not normalize PRL levels (Lo et al., 2017). However, renal transplantation and the subsequent improvement in glomerular filtration result in the normalization of serum PRL levels (Bry-Gauillard et al., 1999; Saha et al., 2002). Studies using the dopaminergic agonists cabergoline and bromocriptine in CKD are scarce. However, when prescribed for clinical conditions such as galactorrhea or hypogonadism, their use is safe (Degli Esposti et al., 1985; Salmela et al., 2006).

Dopaminergic neurons’ functionality and survival are sensitive to cytokines in chronic inflammation conditions (Felger and Miller, 2012). TIDA neurons have estrogen receptors (Mitchell et al., 2003; Ribeiro et al., 2015) and chronic exposure to estradiol affects them, leading to hyperprolactinemia. This initiates an inflammatory cascade in the arcuate nucleus: increased cytokine and nitric oxide production lead to reduced TIDA neuron functioning, which culminates in lower dopamine synthesis and high levels of PRL that increase the risk for mammary and pituitary tumor development (Gilbreath et al., 2019). This process highlights the importance of inflammatory regulators in pathological alterations of the PRL system, by “highjacking” the TIDA circuit and ultimately promoting hyperprolactinemia.

Interestingly, experimental evidence suggests that a hyperprolactinemic state might alter the olfactory function (Corona et al., 2021). Adequate olfactory information processing relies on the precise function of the olfactory bulb (OB), which involves continuous plastic changes that tune the circuit (Shingo et al., 2003; Corona and Levy, 2015). PRL regulates OB neurogenesis, necessary for healthy olfactory functions and behavioral responses (Shingo et al., 2003; Corona and Levy, 2015). Recently, we demonstrated that juvenile hyperprolactinemia alters OB mitral cell activation in female mice exposed to social odors, suggesting that PRL participates in the maturation and response of the OB circuits (Corona et al., 2021). Interestingly, OB mitral cells express PRL receptors (Freemark et al., 1996; Canavan et al., 2011; de Moura et al., 2015), implying that hyperprolactinemia possibly regulates OB functioning and, as a result, the olfactory capacity in CKD.

## Olfactory Function in Disease

### Olfactory Impairments in Disease

Human olfaction involves complex processes that detect, discriminate, and code thousands of odors (McGann, 2017). Olfactory impairments reflect deficient functionality in the periphery with variable odor thresholds or compromised central processing of the olfactory information resulting in diminished odor discrimination and identification (Frasnelli et al., 2002; Raff et al., 2008; Robles-Osorio et al., 2020; Iacono et al., 2021; Yusuf et al., 2021a).

Within the nasal cavity resides the epithelium, where odor molecules bind to the olfactory receptors (Ihara et al., 2013). This information is transduced into a neural signal and sent through the axons of the olfactory sensory neurons (OSN) to synapse the projection mitral or tufted cells, in the glomeruli of the OBs (Martin-Lopez et al., 2012). This first step is essential for olfaction. The OSNs have a lifelong regenerative capacity necessary for adequate olfaction (Lazarini and Lledo, 2011). Reduced tissue maintenance by poor basal stem cell proliferation within the epithelium can generate hyposmia (a significant reduction in olfactory abilities), anosmia (complete loss of olfaction), or olfactory threshold deficiencies due to the lack of neural signals in the OB (Goncalves and Goldstein, 2016; McGann, 2017).

There is a growing interest in olfactory disorders owing to their role as leading indicators in numerous pathologies. Olfactory disorders are associated with many causes like nasal inflammation, upper olfactory tract abnormalities, neurological pathologies, and aging (Huttenbrink et al., 2013; Schriever et al., 2014; Kar et al., 2015). Moreover, olfaction strongly influences human behavior: a wide range of environmental odors primes our safety (e.g., avoiding fire, gas leakage, or rotten food) but also influences eating behavior (e.g., associating memories and emotions with food) and social interactions (McGann, 2017). Initially, olfactory impairments remain unnoticed, forcing the individuals into situations where they must deal with daily issues like feeding, safety, and social situations, which, in many cases, lead to anxiety and depression (Sivam et al., 2016; Kondo et al., 2020). Olfactory deficits promote food aversion and, consequently, malnutrition (Frasnelli et al., 2002).

### Olfactory Function and Inflammation

Inflammatory conditions of the nasal cavity reduce the proliferation of the basal stem cells, while the newly differentiated OSNs do not survive easily (Chen et al., 2019). Inflammation induces OB atrophy by interfering with the input from the OSN axons, reducing the apical dendrites of mitral and tufted cells, and shrinking the glomerular layer thickness (Chen et al., 2019). Also, OB inflammation is accompanied by glial activation and high expression of the cytokines interleukin 1B and tumor necrosis factor α (Hasegawa-Ishii et al., 2020). The basal stem cells directly regulate the inflammatory progression in the olfactory mucosa. In chronic inflammation, the basal cells release cytokines and chemokines to shut down the stem cell proliferation, generating olfactory dysfunctions (Chen et al., 2019).

Damage to the nasal epithelium is reversible when the inflammation is reduced or controlled (Hasegawa-Ishii et al., 2020). This recovery probably explains the discrepancies observed in odor threshold deficiencies, which improve after dialysis treatment (Landis et al., 2011). The complete recovery of odor discrimination and identification after renal transplantation is due to the decreased generalized inflammation that allows for sufficient neuronal information processing (Vreman et al., 1980; Griep et al., 1997; Frasnelli et al., 2002; Landis et al., 2011; Koseoglu et al., 2017; Iacono et al., 2021; Yusuf et al., 2021a). Interestingly, odor stimulation contributes to the recovery of OB atrophy (Hasegawa-Ishii et al., 2020). Olfactory training could serve as a novel strategy and additional treatment in CKD since it has been demonstrated to ameliorate olfactory deficits in aging and various pathologies (Livermore and Hummel, 2004; Hummel et al., 2009; Haehner et al., 2013; Schriever et al., 2014).

### Olfactory Impairments in Chronic Kidney Disease

CKD and particularly ESKD patients present significantly decreased olfactory function (Figure 1; Frasnelli et al., 2002; Landis et al., 2011; Attems et al., 2015; Kondo et al., 2020; Robles-Osorio et al., 2020; Iacono et al., 2021; Yusuf et al., 2021a,b). Additionally, CKD patients follow a controlled diet with reduced calorie uptake, and ∼70% of them suffer from loss of taste (Frasnelli et al., 2002), which can lead to insufficient or unbalanced feeding behaviors. Malnutrition, one of the major causes of morbidity and mortality in CKD (Figure 1; Anderson et al., 2016), begins when renal functions decline, promoting a gradually reduced protein and total calorie intake, leading to cachexia, and increasing the risk of death (Nigwekar et al., 2017; Yusuf et al., 2021b). Although the straightforward link between impaired olfactory function and the nutritional state is still debatable, poor odor perception is associated with elevated serum urea and a high protein catabolic rate (Griep et al., 1997). The pathological changes underlying the malnutrition-inflammatory state may also contribute to impaired olfactory function in ESKD patients (Frasnelli et al., 2002; Raff et al., 2008; Koseoglu et al., 2017; Robles-Osorio et al., 2020; Iacono et al., 2021; Yusuf et al., 2021a).

Up to date, several studies have highlighted the importance of understanding the link between CKD and olfactory sense loss due to its relevance in nutrition and quality of life of the patients (Robles-Osorio et al., 2020; Yusuf et al., 2021a). In recent studies with CKD and ESKD patients, ∼70% presented olfactory dysfunctions, with 72% (Yusuf et al., 2021a) to 83% (Koseoglu et al., 2017) of CKD patients presenting some degree of hyposmia and, in extreme cases, 5% presenting anosmia (Yusuf et al., 2021a).

Renal failure pathologies affect the function of the CNS. In CKD, there is a generalized disequilibrium of biochemical parameters that provokes systemic inflammation (Adesso et al., 2017; Cobo et al., 2018). Odor discrimination and identification are compromised in CKD due to deficient neuronal functioning (Frasnelli et al., 2002). The lack of efficient glomerulus filtration generates endogenous intoxication that damages axons and dendrites, consequently leading to neurodegeneration (Bobot et al., 2020). Furthermore, the impaired blood-brain barrier by the neuropathic uremia promotes changes in the hydration levels within the brain and the cerebral blood flow, altering the synthesis of neurotransmitters (Wiggins et al., 1984; Griep et al., 1997; Burn and Bates, 1998; Frasnelli et al., 2002; Raff et al., 2008; Goncalves and Goldstein, 2016; Nigwekar et al., 2017; Robles-Osorio et al., 2020). It has been consistently shown that CKD and -even more prominently- ESKD patients present deficits in odor discrimination and identification but the odor threshold impairments are less evident or inconsistent across different studies (Schiffman et al., 1978; Griep et al., 1997; Frasnelli et al., 2002; Raff et al., 2008; Landis et al., 2011; Nigwekar et al., 2017; Robles-Osorio et al., 2020; Iacono et al., 2021; Yusuf et al., 2021a).

## Discussion

CKD is characterized by a persistent deficiency in GFR that leads to increased levels of uremic toxins, such as indoxyl sulfate and p-Cresol. The accumulation of uremic toxins promotes the activation of the immune system through the release of cytokines and the lack of clearance of PRL due to inadequate glomerular filtration. CKD patients develop deficiencies in odor detection because of alterations in the olfactory epithelium that modify the odor threshold and are regulated by inflammation. Odor identification and discrimination impairments frequently occur in CKD due to chronic intoxication and inflammation. These olfactory impairments are reversible after renal transplantation. The olfactory alterations promoted by CKD can cause malnutrition, exacerbating the patients’ condition. Evidence on the regulatory role of PRL in olfactory processing suggests an interaction between CKD-induced high PRL levels and the olfactory deficiencies (Figure 1).

In CKD, the deficient elimination of uremic toxins increases the levels of inflammatory cytokines, causing oxidative stress and consequently cellular damage to a variety of systems. CKD is associated with various hormonal alterations. Hyperprolactinemia is a frequent endocrine abnormality in this population as it has been reported in more than 30% of CKD patients (Lo et al., 2017; Serret-Montaya et al., 2020; Franco-Acevedo et al., 2021). PRL levels are directly associated with endothelial dysfunction and increased risk of cardiovascular events and mortality in CKD (Carrero et al., 2012). Although the mechanisms of hyperprolactinemia in CKD are yet to be unraveled, it could be a result of PRL accumulation due to deficient renal clearance. Among the causes of hyperprolactinemia in CKD, the following stand out: (a) the established negative feedback loop in TIDA neurons is “highjacked,” promoting PRL production, (b) estrogens increase the inflammatory response, reducing dopamine production, therefore, enhancing PRL secretion, and (c) renal dysfunction directly alters the PRL metabolic clearance (Sievertsen et al., 1980).

The inflammation-oxidative stress “duo” causes cognitive impairments in CKD patients (Tsuruya and Yoshida, 2018), as the chronic and systemic inflammatory state deteriorates the odor detection at the level of the olfactory epithelium, leading to odor threshold deficiencies which can be reverted after controlling the inflammation (Hasegawa-Ishii et al., 2020). CKD-induced neuropathy compromises odor discrimination and identification, affecting the quality of life and leading to malnutrition and metabolic problems (Frasnelli et al., 2002). In some cases, these alterations are improved after dialysis treatment, while renal transplantation leads to recovery of the olfaction (Robles-Osorio et al., 2020; Yusuf et al., 2021a).

Understanding the link between CKD and the loss of the sense of smell is relevant to the nutritional status and the quality of life of patients. Research in animal models and humans should be directed toward the understanding of the relationship between CKD-induced hyperprolactinemia and the loss or alteration of olfaction. New insights into the timing and progression of the disease and the role of inflammation on neuroendocrine systems, such as PRL, could expand our knowledge and open an avenue toward the improvement of the quality of life in CKD patients. Additionally, the development of a prevention strategy with screening olfactory tests in the early stages of CKD can complement the therapeutical approaches together with the introduction of physical activity and dietary programs.

## Author Contributions

RC, BO, LR-O, ES, and TM wrote and edited the review article. RC and BO created the figure. All authors contributed to the article and approved the submitted version.

## Conflict of Interest

The authors declare that the research was conducted in the absence of any commercial or financial relationships that could be construed as a potential conflict of interest.

## Publisher’s Note

All claims expressed in this article are solely those of the authors and do not necessarily represent those of their affiliated organizations, or those of the publisher, the editors and the reviewers. Any product that may be evaluated in this article, or claim that may be made by its manufacturer, is not guaranteed or endorsed by the publisher.

## References

[B1] Adesso S. Magnus T. Cuzzocrea S. Campolo M. Rissiek B. Paciello O. (2017). Indoxyl sulfate affects glial function increasing oxidative stress and neuroinflammation in chronic kidney disease: interaction between astrocytes and microglia. *Front. Pharmacol.* 8:370. 10.3389/fphar.2017.00370 28659803 PMC5466960

[B2] Aguilar D. J. Madero M. (2019). Other Potential CKD hotspots in the world: the cases of mexico and the united states. *Semin. Nephrol.* 39 300–307. 10.1016/j.semnephrol.2019.02.008 31054630

[B3] Anderson C. A. Nguyen H. A. Rifkin D. E. (2016). Nutrition interventions in chronic kidney disease. *Med. Clin. North Am.* 100 1265–1283. 27745594 10.1016/j.mcna.2016.06.008

[B4] Attems J. Walker L. Jellinger K. A. (2015). Olfaction and aging: a mini-review. *Gerontology* 61 485–490. 10.1159/000381619 25968962

[B5] Ben-Jonathan N. Mershon J. L. Allen D. L. Steinmetz R. W. (1996). Extrapituitary prolactin: distribution, regulation, functions, and clinical aspects. *Endoc. Rev.* 17 639–669. 10.1210/edrv-17-6-639 8969972

[B6] Bhat H. K. Hacker H. J. Bannasch P. Thompson E. A. Liehr J. G. (1993). Localization of estrogen receptors in interstitial cells of hamster kidney and in estradiol-induced renal tumors as evidence of the mesenchymal origin of this neoplasm. *Cancer Res.* 53 5447–5451. 8221684

[B7] Bikbov B. Perico N. Remuzzi G. (2018). Disparities in chronic kidney disease prevalence among males and females in 195 countries: analysis of the global burden of disease 2016 study. *Nephron* 139 313–318. 10.1159/000489897 29791905

[B8] Bobot M. Thomas L. Moyon A. Fernandez S. McKay N. Balasse L. (2020). Uremic toxic blood-brain barrier disruption mediated by ahr activation leads to cognitive impairment during experimental renal dysfunction. *J. Am. Soc. Nephrol.* 31 1509–1521. 10.1681/ASN.2019070728 32527975 PMC7350986

[B9] Borba V. V. Zandman-Goddard G. Shoenfeld Y. (2018). Prolactin and Autoimmunity. *Front. Immunol.* 9:73. 29483903 10.3389/fimmu.2018.00073 PMC5816039

[B10] Bouchard B. Ormandy C. J. Di Santo J. P. Kelly P. A. (1999). Immune system development and function in prolactin receptor-deficient mice. *J. Immunol.* 163 576–582. 10395643

[B11] Brar A. Markell M. (2019). Impact of gender and gender disparities in patients with kidney disease. *Curr. Opin. Nephrol. Hyperten.* 28 178–182. 10.1097/MNH.0000000000000482 30652978

[B12] Bry-Gauillard H. Touraine P. Mamzer-Bruneel M. F. Simoes-Vaz A. Kuttenn F. Legendre C. (1999). Complete regression of a major hyperprolactinaemia after renal transplantation. *Nephrol. Dial. Transpl.* 14 466–468. 10.1093/ndt/14.2.466 10069216

[B13] Bugnicourt J. M. Godefroy O. Chillon J. M. Choukroun G. Massy Z. A. (2013). Cognitive disorders and dmentia in CKD: the neglected kidney-brain-axis. *J. Am. Soc. Nephrol.* 24 353–363. 10.1681/ASN.2012050536 23291474

[B14] Burn D. J. Bates D. (1998). Neurology and the kidney. *J. Neurol. Neurosurg. Psychiatry* 65 810–821. 10.1136/jnnp.65.6.810 9854955 PMC2170399

[B15] Canavan S. V. Mayes L. C. Treloar H. B. (2011). Changes in maternal gene expression in olfactory circuits in the immediate postpartum period. *Front. psychiatry* 2:40. 10.3389/fpsyt.2011.00040 21747772 PMC3130163

[B16] Carrero J. J. Hecking M. Chesnaye N. C. Jager K. J. (2018). Sex and gender disparities in epidemiology and outcomes of chronic kidney disease. *Nat. Rev. Nephrol.* 14 151–164. 10.1038/nrneph.2017.181 29355169

[B17] Carrero J. J. Kyriazis J. Sonmez A. Tzanakis I. Qureshi A. R. Stenvinkel P. (2012). Prolactin levels, endothelial dysfunction, and the risk of cardiovascular events and mortality in patients with CKD. *Clin. J. Am. Soc. Nephrol.* 7 207–215. 10.2215/CJN.06840711 22193237 PMC3280028

[B18] Chen M. Reed R. R. Lane A. P. (2019). Chronic inflammation directs an olfactory stem cell functional switch from neuroregeneration to immune defense. *Cell Stem cell* 25:e505. 10.1016/j.stem.2019.08.011 31523027 PMC6778045

[B19] Cheng T. H. Ma M. C. Liao M. T. Zheng C. M. Lu K. C. Liao C. H. (2020). Indoxyl sulfate, a tubular toxin, contributes to the development of chronic kidney disease. *Toxins* 12:684. 10.3390/toxins12110684 33138205 PMC7693919

[B20] Cigarran Guldris S. GonzalezParra E. CasesAmenos A. (2017). Gut microbiota in chronic kidney disease. *Nefrologia : Publicacion Oficial De La Sociedad Espanola Nefrologia* 37 9–19. 27553986 10.1016/j.nefro.2016.05.008

[B21] Cobo G. Lindholm B. Stenvinkel P. (2018). Chronic inflammation in end-stage renal disease and dialysis. *Nephrol. Dial Transpl.* 33 iii35–iii40. 10.1093/ndt/gfy175 30281126 PMC6168801

[B22] Cockwell P. Fisher L. A. (2020). The globan burden of chronic kidney disease. *Lancet* 395 662–664. 32061314 10.1016/S0140-6736(19)32977-0

[B23] Corona R. Jayakumar P. Carbajo Mata M. A. Del Valle-Diaz M. F. Luna-Garcia L. A. Morales T. (2021). Sexually dimorphic effects of prolactin treatment on the onset of puberty and olfactory function in mice. *Gener. Compar. Endocrinol.* 301:113652. 10.1016/j.ygcen.2020.113652 33122037

[B24] Corona R. Levy F. (2015). Chemical olfactory signals and parenthood in mammals. *Hormon. Behav.* 68 77–90. 10.1016/j.yhbeh.2014.06.018 25038290

[B25] Dalla Valle L. Toffolo V. Vianello S. Belvedere P. Colombo L. (2004). Expression of cytochrome P450c17 and other steroid-converting enzymes in the rat kidney throughout the life-span. *J. Ster. Biochem. Mol. Biol.* 91 49–58. 10.1016/j.jsbmb.2004.01.008 15261307

[B26] Davidoff M. Caffier H. Schiebler T. H. (1980). Steroid hormone binding receptors in the rat kidney. *Histochemistry* 69 39–48. 10.1007/BF00508365 7440259

[B27] de Moura A. C. Lazzari V. M. Becker R. O. Gil M. S. Ruthschilling C. A. Agnes G. (2015). Gene expression in the CNS of lactating rats with different patterns of maternal behavior. *Neurosci. Res.* 99 8–15. 10.1016/j.neures.2015.05.003 26003743

[B28] Degli Esposti E. Sturani A. Santoro A. Zuccala A. Chiarini C. Zucchelli P. (1985). Effect of bromocriptine treatment on prolactin, noradrenaline and blood pressure in hypertensive haemodialysis patients. *Clin. Sci.* 69 51–56. 10.1042/cs0690051 3905210

[B29] Demaria J. E. Nagy G. M. Lerant A. A. Fekete M. I. Levenson C. W. Freeman M. E. (2000). Dopamine transporters participate in the physiological regulation of prolactin. *Endocrinology* 141 366–374. 10.1210/endo.141.1.7281 10614659

[B30] Dhondup T. Qian Q. (2017). Acid-Base and electrolyte disorders in patients with and without chronic kidney disease: an update. *Kidney Dis.* 3 136–148. 10.1159/000479968 29344508 PMC5757582

[B31] Dourado M. Cavalcanti F. Vilar L. Cantilino A. (2020). Relationship between prolactin, chronic kidney disease, and cardiovascular risk. *Int. J. Endocrinol.* 2020 1–6 10.1155/2020/9524839 32655635 PMC7327580

[B32] Emmanouel D. S. Fang V. S. Katz A. I. (1981). Prolactin metabolism in the rat: role of the kidney in degradation of the hormone. *Am. J. Physiol.* 240 F437–F445. 10.1152/ajprenal.1981.240.5.F437 7235018

[B33] Felger J. C. Miller A. H. (2012). Cytokine effects on the basal ganglia and dopamine function: the subcortical source of inflammatory malaise. *Front. Neuroendocrinol.* 33:315–327. 10.1016/j.yfrne.2012.09.003 23000204 PMC3484236

[B34] Franco-Acevedo A. Echavarría R. Melo Z. (2021). Sex differences in renal function: participation of gonadal hormones and prolactin. *Endocrines* 2 185–202. 10.1016/j.envint.2017.10.006 29055783

[B35] Frasnelli J. A. Temmel A. F. Quint C. Oberbauer R. Hummel T. (2002). Olfactory function in chronic renal failure. *Am. J. Rhinol.* 16 275–279. 10.1177/194589240201600511 12422973

[B36] Freeman M. E. Kanyicska B. Lerant A. Nagy G. (2000). Prolactin: structure, function, and regulation of secretion. *Physiol. Rev.* 80 1523–1631. 10.1152/physrev.2000.80.4.1523 11015620

[B37] Freemark M. Driscoll P. Andrews J. Kelly P. A. Royster M. (1996). Ontogenesis of prolactin receptor gene expression in the rat olfactory system: potential roles for lactogenic hormones in olfactory development. *Endocrinology* 137 934–942. 10.1210/endo.137.3.8603606 8603606

[B38] Gilbreath E. T. Jaganathan L. Subramanian M. Balasubramanian P. Linning K. D. MohanKumar S. M. J. (2019). Chronic estrogen affects TIDA neurons through IL-1beta and NO: effects of aging. *J. Endocrinol.* 240 157–167. 10.1530/JOE-18-0274 30400030 PMC13321404

[B39] Goncalves S. Goldstein B. J. (2016). Pathophysiology of olfactory disorders and potential treatment strategies. *Curr. Otorhinolaryngol. Rep.* 4 115–121. 10.1007/s40136-016-0113-5 27529054 PMC4982703

[B40] Grattan D. R. Xu J. McLachlan M. J. Kokay I. C. Bunn S. J. Hovey R. C. (2001). Feedback regulation of PRL secretion is mediated by the transcription factor, signal transducer, and activator of transcription 5b. *Endocrinology* 142 3935–3940. 10.1210/endo.142.9.8385 11517172

[B41] Griep M. I. Van der Niepen P. Sennesael J. J. Mets T. F. Massart D. L. Verbeelen D. L. (1997). Odour perception in chronic renal disease. *Nephrol. Dial. Transpl.* 12 2093–2098. 10.1093/ndt/12.10.2093 9351071

[B42] Gungor O. Kircelli F. Voroneanu L. Covic A. Ok E. (2013). Hormones and arterial stiffness in patients with chronic kidney disease. *J. Atheroscl. Thromb.* 20 698–707. 10.5551/jat.18580 23911970

[B43] Gutierrez-Padilla J. A. Mendoza-García M. Plascencia-Pérez S. Renoirte-López K. García-García G. Lloyd A. (2010). Screening for CKD and cardiovascular disease risk factors using mobile clinics in Jalisco, México. *Am. J. Kidney Dis.* 55 474–484. 10.1053/j.ajkd.2009.07.023 19850389

[B44] Haehner A. Tosch C. Wolz M. Klingelhoefer L. Fauser M. Storch A. (2013). Olfactory training in patients with Parkinson’s disease. *PloS One* 8:e61680. 10.1371/journal.pone.0061680 23613901 PMC3629137

[B45] Han H. J. Park S. H. Park H. J. Lee J. H. Lee B.-C. Hwang W. S. (2001). Effects of Sex Hormones on Na+/Glucose cotransporter of renal proximal tubular cells following oxidant injury. *Kidney Blood Press Res.* 24 159–165. 10.1159/000054223 11528208

[B46] Hasegawa-Ishii S. Imamura F. Nagayama S. Murata M. Shimada A. (2020). Differential effects of nasal inflammation and odor deprivation on layer-specific degeneration of the mouse olfactory bulb. *eNeuro* 7:2020. 10.1523/ENEURO.0403-19.2020 32220858 PMC7168263

[B47] Horrobin D. F. Lloyd I. J. Lipton A. Burstyn P. G. Durkin N. Muiruri K. L. (1971). Actions of prolactin on human renal function. *Lancet* 2 352–354. 10.1016/s0140-6736(71)90065-1 4105050

[B48] Hummel T. Rissom K. Reden J. Hahner A. Weidenbecher M. Huttenbrink K. B. (2009). Effects of olfactory training in patients with olfactory loss. *Laryngoscope* 119 496–499. 10.1002/lary.20101 19235739

[B49] Huttenbrink K. B. Hummel T. Berg D. Gasser T. Hahner A. (2013). Olfactory dysfunction: common in later life and early warning of neurodegenerative disease. *Deutsch. Arzteblatt Int.* 110:e1. 10.3238/arztebl.2013.0001 23450985 PMC3561743

[B50] Iacono V. Lombardi G. Ottaviano G. Gambaro G. Zaza G. (2021). Impact of renal replacement therapies on olfactory ability: results of a cross-sectional case control study. *J. Nephrol.* 2021:983. 10.1007/s40620-021-00983-6 33625692 PMC8803626

[B51] Ihara S. Yoshikawa K. Touhara K. (2013). Chemosensory signals and their receptors in the olfactory neural system. *Neuroscience* 254 45–60. 10.1016/j.neuroscience.2013.08.063 24045101

[B52] Ji-Cheng L. Lu-Xia Z. (2019). Prevalence and disease burden of chronic kidney disease. *Adv. Exp. Med. Biol.* 1165 3–15. 10.1007/978-981-13-8871-2_1 31399958

[B53] Kang K. P. Lee J. E. Lee A. S. Jung Y. J. Kim D. Lee S. (2014). Effect of gender differences on the regulation of renal ischemia-reperfusion-induced inflammation in mice. *Mol. Med. Rep.* 9 2061–2068. 10.3892/mmr.2014.2089 24682292 PMC4055478

[B54] Kar T. Yildirim Y. Altundag A. Sonmez M. Kaya A. Colakoglu K. (2015). The relationship between age-related macular degeneration and olfactory function. *Neuro-Degener. Dis.* 15 219–224. 10.1159/000381216 25871947

[B55] Karbowska M. Hermanowicz J. M. Tankiewicz-Kwedlo A. Kalaska B. Kaminski T. W. Nosek K. (2020). Neurobehavioral effects of uremic toxin-indoxyl sulfate in the rat model. *Sci. Rep.* 10:9483. 10.1038/s41598-020-66421-y 32528183 PMC7289875

[B56] Kondo K. Kikuta S. Ueha R. Suzukawa K. Yamasoba T. (2020). Age-related olfactory dysfunction: epidemiology, pathophysiology, and clinical management. *Front. Aging Neurosci.* 12:208. 10.3389/fnagi.2020.00208 32733233 PMC7358644

[B57] Koseoglu S. Derin S. Huddam B. Sahan M. (2017). The effect of non-diabetic chronic renal failure on olfactory function. *Eur. Ann. Otorhinolaryngol. Head Neck Dis.* 134 161–164. 10.1016/j.anorl.2016.04.022 27988196

[B58] Krajewska J. Krajewski W. Zatonski T. (2020). Otorhinolaryngological dysfuntions induced by chronic kidney disease in pre- and post-transplant stages. *Eur. Arch. Oto-Rhino-Laryngol.* 277 1575–1591. 10.1007/s00405-020-05925-9 32222803 PMC7198632

[B59] Landis B. N. Marangon N. Saudan P. Hugentobler M. Giger R. Martin P. Y. (2011). Olfactory function improves following hemodialysis. *Kidney Int*, 80 886–893. 10.1038/ki.2011.189 21697812

[B60] Lazarini F. Lledo P. M. (2011). Is adult neurogenesis essential for olfaction? *Trends Neurosci.* 34 20–30. 10.1016/j.tins.2010.09.006 20980064

[B61] Leite De Moraes M. C. Touraine P. Gagnerault M. C. Savino W. Kelly P. A. Dardenne M. (1995). Prolactin receptors and the immune system. *Ann. Endocrinol.* 56 567–570. 8787345

[B62] Levey A. S. Eckardt K. U. Dorman N. M. Christiansen S. L. Hoorn E. J. Ingelfinger J. R. (2020). Nomenclature for kidney function and disease: report of a kidney disease: improving global outcomes (KDIGO) Consensus Conference. *Kidney Int.* 97 1117–1129. 10.1016/j.kint.2020.02.010 32409237

[B63] Lima-Posada I. Bobadilla N. A. (2021). Understanding the opposite effects of sex hormones in mediating renal injury. *Nephrology* 26 217–226. 10.1111/nep.13806 33058388

[B64] Lima-Posada I. Portas-Cortes C. Perez-Villalva R. Fontana F. Rodriguez-Romo R. Prieto R. (2017). Gender differences in the acute kidney injury to chronic kidney disease transition. *Sci. Rep.* 7:12270. 10.1038/s41598-017-09630-2 28947737 PMC5612964

[B65] Livermore A. Hummel T. (2004). The influence of training on chemosensory event-related potentials and interactions between the olfactory and trigeminal systems. *Chem. Senses* 29 41–51. 10.1093/chemse/bjh013 14752039

[B66] Lo J. C. Beck G. J. Kaysen G. A. Chan C. T. Kliger A. S. Rocco M. V. (2017). Hyperprolactinemia in end-stage renal disease and effects of frequent hemodialysis. *Hemodial. Int.* 21 190–196. 10.1111/hdi.12489 27774730 PMC7481141

[B67] Loretz C. A. Bern H. A. (1982). Prolactin and osmoregulation in vertebrates. An update. *Neuroendocrinology* 35 292–304. 10.1159/000123397 6292764

[B68] Ma F. Y. Anderson G. M. Gunn T. D. Goffin V. Grattan D. R. Bunn S. J. (2005). Prolactin specifically activates signal transducer and activator of transcription 5b in neuroendocrine dopaminergic neurons. *Endocrinology* 146 5112–5119. 10.1210/en.2005-0770 16123156

[B69] Machluf Y. Chaiter Y. Tal O. (2020). Gender medicine: Lessons from COVID-19 and other medical conditions for designing health policy. *World J. Clin. Cases* 8 3645–3668. 10.12998/wjcc.v8.i17.3645 32953842 PMC7479575

[B70] Martin-Lopez E. Corona R. Lopez-Mascaraque L. (2012). Postnatal characterization of cells in the accessory olfactory bulb of wild type and reeler mice. *Front. Neuroanat.* 6:15. 10.3389/fnana.2012.00015 22661929 PMC3357593

[B71] Martucci C. P. Fishmann J. (1993). P450 enzyme of estrogen metabolism. *Pharmacol. Ther.* 57 237–257. 8361994 10.1016/0163-7258(93)90057-k

[B72] McGann J. P. (2017). Poor human olfaction is a 19th-century myth. *Science* 356:7263. 10.1126/science.aam7263 28495701 PMC5512720

[B73] Mihai S. Codrici E. Popescu I. D. Enciu A. M. Albulescu L. Necula L. G. (2018). Inflammation-related mechanisms in chronic kidney disease prediction, progression, and outcome. *J. Immunol. Res.* 2018:2180373. 10.1155/2018/2180373 30271792 PMC6146775

[B74] Miletić D. Fuckar Z. Sustić A. Mozetic V. Stimac D. Zauhar G. (1998). Sonographic measurement of absolute and relative renal length in adults. *J. Clin. Ultrasound* 26 185–189. 10.1002/(sici)1097-0096(199805)26:4<185::aid-jcu1>3.0.co;2-9 9572380

[B75] Mills K. T. Xu Y. Zhang W. Bundy J. D. Chen C. S. Kelly T. N. (2015). A systematic analysis of worldwide population-based data on the global burden of chronic kidney disease in 2010. *Kidney Int.* 88 950–957. 10.1038/ki.2015.230 26221752 PMC4653075

[B76] Mitchell V. Loyens A. Spergel D. J. Flactif M. Poulain P. Tramu G. (2003). A confocal microscopic study of gonadotropin-releasing hormone (GnRH) neuron inputs to dopaminergic neurons containing estrogen receptor alpha in the arcuate nucleus of GnRH-green fluorescent protein transgenic mice. *Neuroendocrinology* 77 198–207. 10.1159/000069511 12673053

[B77] Morales T. (2011). Recent findings on neuroprotection against excitotoxicity in the hippocampus of female rats. *J. Neuroendocrinol.* 23 994–1001. 10.1111/j.1365-2826.2011.02141.x 21507086

[B78] Neugarten J. Kasiske B. Silbiger S. R. Nyengaard J. R. (2002). Effects of sex on renal structure. *Nephron* 90 139–144. 10.1159/000049033 11818696

[B79] Nigwekar S. U. Weiser J. M. Kalim S. Xu D. Wibecan J. L. Dougherty S. M. (2017). Characterization and correction of olfactory deficits in kidney disease. *J. Am. Soc. Nephrol.* 28 3395–3403. 10.1681/ASN.2016121308 28775001 PMC5661283

[B80] No Author. (2013). Chapter 1: Definition and classification of CKD. *Kidney Int. Suppl.* 3 19–62. 10.1038/kisup.2012.64 25018975 PMC4089693

[B81] Olano C. G. Akram S. M. Bhatt H. (2021). *Uremic Encephalopathy.* Treasure Island, FL: StatPearls. 33231997

[B82] Opdebeeck B. Maudsley S. Azmi A. De Mare A. De Leger W. Meijers B. (2019). Indoxyl Sulfate and p-Cresyl sulfate promote vascular calcification and associate with glucose intolerance. *J. Am. Soc. Nephrol.* 30 751–766. 10.1681/ASN.2018060609 30940651 PMC6493976

[B83] Quinkler M. Bumke-Vogt C. Meyer B. Bahr V. Oelkers W. Diederich S. (2003). The human kidney is a progesterone-metabolizing and androgen-producing organ. *J Clin. Endocrinol. Metabol.* 88 2803–2809. 10.1210/jc.2002-021970 12788891

[B84] Raff A. C. Lieu S. Melamed M. L. Quan Z. Ponda M. Meyer T. W. (2008). Relationship of impaired olfactory function in ESRD to malnutrition and retained uremic molecules. *Am. J. Kidney Dis.* 52 102–110. 10.1053/j.ajkd.2008.02.301 18423810 PMC2712939

[B85] Ribeiro A. B. Leite C. M. Kalil B. Franci C. R. Anselmo-Franci J. A. Szawka R. E. (2015). Kisspeptin regulates tuberoinfundibular dopaminergic neurones and prolactin secretion in an oestradiol-dependent manner in male and female rats. *J. Neuroendocrinol.* 27 88–99. 10.1111/jne.12242 25453900

[B86] Robles-Osorio M. L. Corona R. Morales T. Sabath E. (2020). Chronic kidney disease and the olfactory system. *Nefrologia* 40 120–125. 10.1016/j.nefro.2019.04.009 31371033

[B87] Rossaint J. Oehmichen J. Van Aken H. Reuter S. Pavenstadt H. J. Meersch M. (2016). FGF23 signaling impairs neutrophil recruitment and host defense during CKD. *J. Clin. Invest.* 126 962–974. 10.1172/JCI83470 26878171 PMC4767336

[B88] Rysz J. Franczyk B. Lawinski J. Olszewski R. Cialkowska-Rysz A. Gluba-Brzozka A. (2021). The Impact of CKD on Uremic Toxins and Gut Microbiota. *Toxins* 13:252. 10.3390/toxins13040252 33807343 PMC8067083

[B89] Sabolic I. Asif A. R. Budach W. E. Wanke C. Bahn A. Burckhardt G. (2007). Gender differences in kidney function. *Pflug. Archiv.* 455 397–429. 10.1016/b978-012440905-7/50305-4 17638010

[B90] Safarpour Y. Vaziri N. D. Jabbari B. (2020). Movement disorders in chronic kidney disease - a descriptive review. *J. Stroke Cerebrovascul. Dis.* 30:105408. 10.1016/j.jstrokecerebrovasdis.2020.105408 33139171

[B91] Saha M. T. Saha H. H. Niskanen L. K. Salmela K. T. Pasternack A. I. (2002). Time course of serum prolactin and sex hormones following successful renal transplantation. *Nephron* 92 735–737. 10.1159/000064079 12372970

[B92] Sakai Y. Hiraoka Y. Ogawa M. Takeuchi Y. Aiso S. (1999). The prolactin gene is expressed in the mouse kidney. *Kidney Int.* 55 833–840. 10.1046/j.1523-1755.1999.055003833.x 10027920

[B93] Salmela O. Li L. Mamputu J. C. Beauchamp F. Maingrette F. Renier G. (2006). The influences of hyperprolactinemia and obesity on cardiovascular risk markers: effects of cabergoline therapy. *Clin. Endocrinol.* 64 366–370. 10.1111/j.1365-2265.2006.02469.x 16584506

[B94] Schiffman S. S. Nash M. L. Dackis C. (1978). Reduced olfactory discrimination in patients on chronic hemodialysis. *Physiol. Behav.* 21 239–242. 10.1016/0031-9384(78)90046-x 693648

[B95] Schriever V. A. Lehmann S. Prange J. Hummel T. (2014). Preventing olfactory deterioration: olfactory training may be of help in older people. *J. Am. Geriat. Soc.* 62 384–386. 10.1111/jgs.12669 24521370

[B96] Serret-Montaya J. Zurita-Cruz J. N. Villasis-Keever M. A. Aguilar-Kitsu A. Zepeda-Martinez C. C. Cruz-Anleu I. (2020). Hyperprolactinemia as a prognostic factor for menstrual disorders in female adolescents with advanced chronic kidney disease. *Pediatric Nephrol.* 35 1041–1049. 10.1007/s00467-020-04494-7 32040631

[B97] Sharpe R. M. (1998). The roles of oestrogen in the male. *Trends Endocrinol. Metabol.* 9 371–377. 10.1016/s1043-2760(98)00089-7 18406308

[B98] Shingo T. Gregg C. Enwere E. Fujikawa H. Hassam R. Geary C. (2003). Pregnancy-stimulated neurogenesis in the adult female forebrain mediated by prolactin. *Science* 299 117–120. 10.1126/science.1076647 12511652

[B99] Sievertsen G. D. Lim V. S. Nakawatase C. Frohman L. A. (1980). Metabolic clearance and secretion rates of human prolactin in normal subjects and in patients with chronic renal failure. *J. Clin. Endocrinol. Metabol.* 50 846–852. 10.1210/jcem-50-5-846 7372775

[B100] Sivam A. Wroblewski K. E. Alkorta-Aranburu G. Barnes L. L. Wilson R. S. Bennett D. A. (2016). Olfactory dysfunction in older adults is associated with feelings of depression and loneliness. *Chem. Senses* 41 293–299. 10.1093/chemse/bjv088 26809485 PMC5006107

[B101] Stier C. T. Jr. Cowden E. A. Friesen H. G. Allison M. E. (1984). Prolactin and the rat kidney: a clearance and micropuncture study. *Endocrinology* 115 362–367. 10.1210/endo-115-1-362 6734519

[B102] Takeda H. Chodak G. Mutchnik S. Nakamoto T. Chang C. (1990). Immunohistochemical localization of androgen receptors with mono- and polyclonal antibodies to androgen receptor. *J. Endocrinol.* 126 17–25. 10.1677/joe.0.1260017 2199591

[B103] Tinti F. Lai S. Noce A. Rotondi S. Marrone G. Mazzaferro S. (2021). Chronic kidney disease as a systemic inflammatory syndrome: update on mechanisms involved and potential treatment. *Life* 11:419. 10.3390/life11050419 34063052 PMC8147921

[B104] Tsuruya K. Yoshida H. (2018). Brain athrophy and cognitive impairment in chronic kidney disease. *Contrib. Nephrol.* 196 27–36. 30041201 10.1159/000485694

[B105] Vanholder R. Pletinck A. Schepers E. Glorieux G. (2018). Biochemical and clinical impact of organic uremic retention solutes: a comprehensive update. *Toxins* 10:33. 10.3390/toxins10010033 29316724 PMC5793120

[B106] Vreman H. J. Venter C. Leegwater J. Oliver C. Weiner M. W. (1980). Taste, smell and zinc metabolism in patients with chronic renal failure. *Nephron* 26 163–170. 10.1159/000181974 7432578

[B107] Watanabe H. Miyamoto Y. Honda D. Tanaka H. Wu Q. Endo M. (2013). p-Cresyl sulfate causes renal tubular cell damage by inducing oxidative stress by activation of NADPH oxidase. *Kidney Int.* 83 582–592. 10.1038/ki.2012.448 23325087

[B108] Watanabe K. Watanabe T. Nakayama M. (2014). Cerebro-renal interactions: impact of uremic toxins on cognitive function. *Neurotoxicology* 44 184–193. 10.1016/j.neuro.2014.06.014 25003961

[B109] Wiggins R. C. Fuller G. Enna S. J. (1984). Undernutrition and the development of brain neurotransmitter systems. *Life Sci.* 35 2085–2094. 10.1016/0024-3205(84)90507-1 6149444

[B110] Yusuf T. Raji Y. R. Daniel A. Bamidele O. T. Fasunla A. J. Lasisi O. A. (2021a). Effect of chronic kidney disease on olfactory function: a case-control study. *Ear Nose Throat J.* 2021:145561321996628. 10.1177/0145561321996628 33617293

[B111] Yusuf T. Raji Y. R. Lasisi T. J. Daniel A. Bamidele O. T. Fasunla A. J. (2021b). Predictors of taste dysfunction and its severity among patients with chronic kidney disease. *Ear Nose Throat J.* 2021:1455613211019708. 10.1177/01455613211019708 34281407

